# Long non-coding RNA ZFAS1 is a major regulator of epithelial-mesenchymal transition through miR-200/ZEB1/E-cadherin, vimentin signaling in colon adenocarcinoma

**DOI:** 10.1038/s41420-021-00427-x

**Published:** 2021-03-26

**Authors:** Stephen J. O’Brien, Casey Fiechter, James Burton, Jacob Hallion, Mason Paas, Ankur Patel, Ajay Patel, Andre Rochet, Katharina Scheurlen, Sarah Gardner, Maurice Eichenberger, Harshini Sarojini, Sudhir Srivastava, Shesh Rai, Theodore Kalbfleisch, Hiram C. Polk, Susan Galandiuk

**Affiliations:** 1 Price Institute of Surgical Research, Hiram C. Polk Jr. MD Department of Surgery, University of Louisville School of Medicine, Louisville, KY 40202 USA; 2 Department of Bioinformatics and Biostatistics, University of Louisville, Louisville, KY USA; 3 Centre for Agricultural Bioinformatics, ICAR-Indian Agricultural Statistics Research Institute, New Delhi, India; 4 Department of Veterinary Science, Gluck Equine Research Center, University of Kentucky, Lexington, KY USA

**Keywords:** Colon cancer, Metastasis, Long non-coding RNAs, Epithelial-mesenchymal transition, Mechanisms of disease

## Abstract

Colon adenocarcinoma is a common cause of cancer-related deaths worldwide. Epithelial-mesenchymal transition is a major regulator of cancer metastasis, and increased understanding of this process is essential to improve patient outcomes. Long non-coding RNA (lncRNA) are important regulators of carcinogenesis. To identify lncRNAs associated with colon carcinogenesis, we performed an exploratory differential gene expression analysis comparing paired colon adenocarcinoma and normal colon epithelium using an RNA-sequencing data set. This analysis identified lncRNA ZFAS1 as significantly increased in colon cancer compared to normal colon epithelium. This finding was validated in an institutional cohort using laser capture microdissection. ZFAS1 was also found to be principally located in the cellular cytoplasm. ZFAS1 knockdown was associated with decreased cellular proliferation, migration, and invasion in two colon cancer cell lines (HT29 and SW480). MicroRNA-200b and microRNA-200c (miR-200b and miR-200c) are experimentally validated targets of ZFAS1, and this interaction was confirmed using reciprocal gene knockdown. ZFAS1 knockdown regulated ZEB1 gene expression and downstream targets E-cadherin and vimentin. Knockdown of miR-200b or miR-200c reversed the effect of ZFAS1 knockdown in the ZEB1/E-cadherin, vimentin signaling cascade, and the effects of cellular migration and invasion, but not cellular proliferation. ZFAS1 knockdown was also associated with decreased tumor growth in an in vivo mouse model. These results demonstrate the critical importance of ZFAS1 as a regulator of the miR-200/ZEB1/E-cadherin, vimentin signaling cascade.

## Introduction

Colorectal cancer is a major cause of cancer-related death worldwide^1^. Although large cohort studies demonstrate that early detection with screening is associated with earlier stage at diagnosis and improved survival, many patients still present with advanced stage (Stage III or IV) disease^2^. Increased understanding of the complex processes involved in tumor metastasis are critical to identifying potential treatment targets and improving patient outcomes.

Epithelial-mesenchymal transition (EMT) is a normal part of embryological development but is also a well characterized and critical part of tumor metastasis^3^. Colorectal cancers that demonstrate a mesenchymal phenotype have worse clinical outcomes compared to those with an epithelial phenotype^4^. EMT is a reversible process whereby cells lose key morphological and phenotypic epithelial features and gain mesenchymal features. The process is regulated by both genetic and epigenetic factors, such as non-coding RNA regulation. Elucidation of such factors specific to colorectal cancer are needed to develop more sophisticated screening tools and targeted therapies, thereby improving patient outcomes.

Long-non coding RNA (lncRNA) are a class of molecules that have recently been described to have a major role in cancer signaling^5^. They mediate their effect through a number of different mechanisms of action. The molecular decoy, or competitive endogenous RNA mechanism, is frequently described. With this mechanism, lncRNAs can competitively bind molecules such as microRNA (miRNA), and prevent them from mediating their effect on downstream gene signaling. ZFAS1 is a lncRNA on chromosome 20 and has been examined in different cancers but variable results have been reported^6^. A recent review described that the majority of studies report that ZFAS1 has an oncogenic role, but ZFAS1 has a tumor suppressor role in breast cancer, and conflicting mechanisms have been reported in hepatocellular cancer^6^. It is important to examine the role of lncRNAs in individual cancer types. The miR-200 family is a well characterized regulator of EMT in cancer that has been demonstrated in a number of in vitro and human studies^4^. The miR-200 family is a group of five miRNAs and its core mechanism is regulated through a double-negative feedback loop with ZEB1/ZEB2 expression^7^.

In this study, we describe the identification and validation of lncRNA ZFAS1 as having an important role in colon cancer progression using a large RNA-seq data set and institutional clinical samples. ZFAS1 was increased in expression in CRC tissue compared to normal colon epithelium across all data sets examined. ZFAS1 knockdown was associated with decreased cellular proliferation, migration, and invasion. In examining the mechanism of action, ZFAS1 was able to regulate Zinc-finger E-box binding homeobox 1 (ZEB1) expression by altering miR-200b and miR-200c signaling. Therefore, ZFAS1 may be a potential future therapeutic target.

## Results

### ZFAS1 is a highly differentially expressed lncRNA in colon adenocarcinoma compared to normal colon epithelium

Using the colon adenocarcinoma TCGA data set, we identified 40 patients with paired colon adenocarcinoma and normal colon epithelium RNA-seq data files. A differential gene expression analysis was performed to identify dysregulated lncRNAs between the paired cancer and normal colon epithelium samples. Using a log fold change of >1.5 or < −1.5, and a false discovery rate <0.05, 348 lncRNAs were found to be differentially expressed (Supplementary Fig. 1).

To narrow the lncRNAs for further investigation, the Cancer Cell Line Encyclopedia was interrogated for colon cancer cell lines with robust lncRNA expression^8^. lncRNAs were also selected on the basis of having experimentally validated lncRNA-miRNA interactions on the DIANA lncBASE platform, as well as reports in the literature^9^. From this, seven lncRNA were selected for further investigation: FAM83H-AS1, PVT1, UCA1, H19, FER1L4, GAS5, and ZFAS1.

**Fig. 1:**
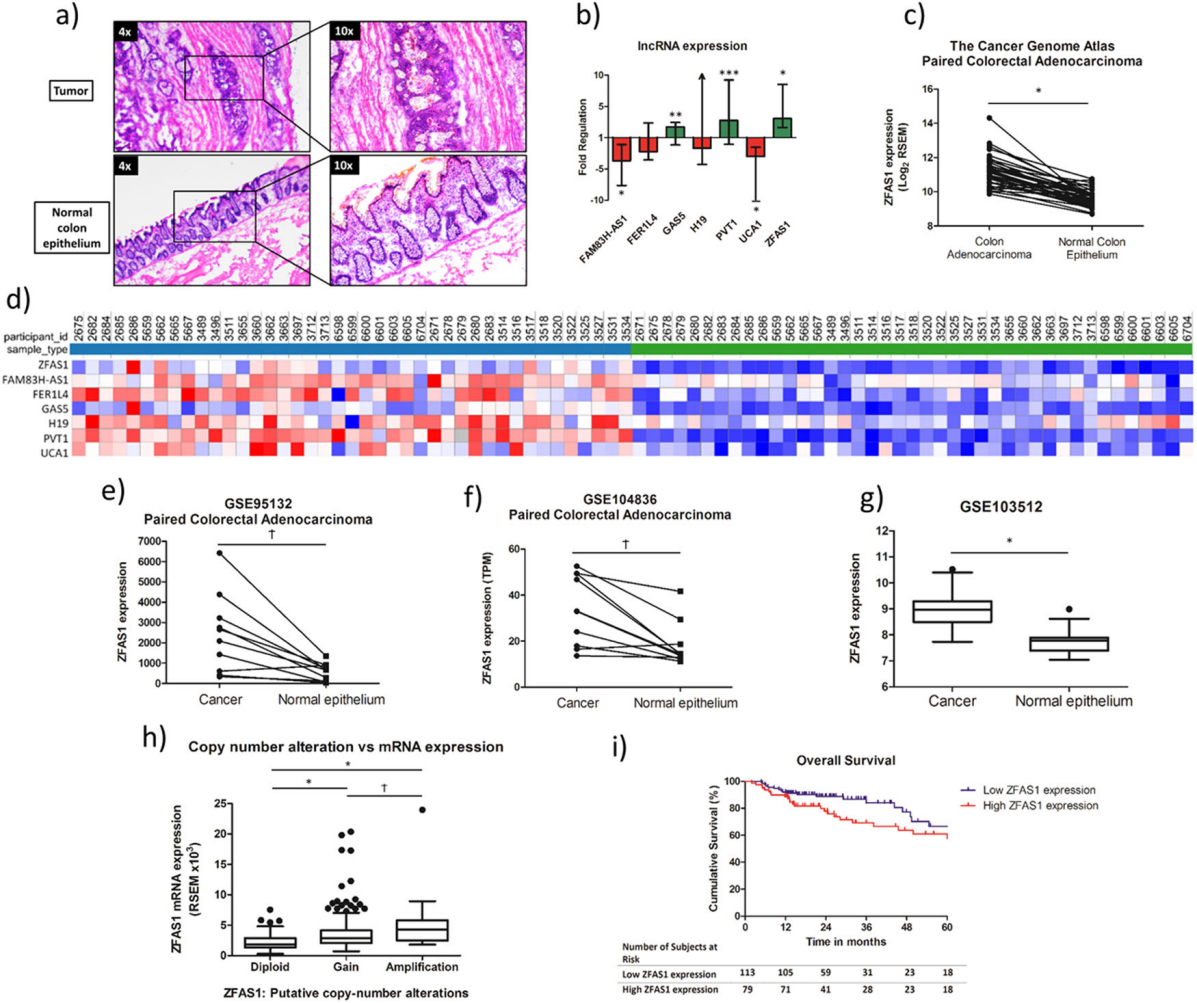
Characterization of lncRNA expression in the TCGA data set and in an institutional data set, with a specific focus on ZFAS1. **a** Example of a hematoxylin and eosin slide that was used as a guide for laser capture microdissection of the tissue from the University of Louisville Biorepository. **b** Fold regulaton of each of the seven selected lncRNAs examined by RTqPCR from LCM tissue from the University of Louisville Biorepository; FAM83H-AS1 (FR = −3.68, *p < 0.001), FER1L4 (FR = −2.22 *p* = 0.12), GAS5 (FR = 1.70, **p = 0.021), H19 (FR = −1.67, *p* = 0.84), PVT1 (FR = 2.75, ***p = 0.003), UCA1 (FR = −4.85, *p < 0.001), ZFAS1 (FR = 3.06, *p < 0.001), (*n* = 23 paired samples). **c** Scatter plot of ZFAS1 expression in colon cancer compared to paired normal colon epithelium examined by RTqPCR from LCM tissue from the University of Louisville Biorepository (*n* = 23 paired institutional samples) (FC = 3.06, *p < 0.001). **d** Heatmap comparing the differential expression (RSEM quantification) between paired colon adenocarcinoma and normal colon epithelium of the seven lncRNAs using RNAseq expression data from the Cancer Genome Atlas (*n* = 40 paired samples). **e** Increased ZFAS1 expression in cancer compared to paired normal colon epithelium in the GEO GSE95132 RNA-seq dataset (*n* = 10 paired samples, RNA-seq expression, units=TPM) (^Ϯ^*p* = 0.009). **f** Increased expression of ZFAS1 in cancer compared to paired normal colon epithelium in the GEO GSE104836 RNA-seq dataset (*n* = 10 paired samples, units = normalized RNAseq expression) (^Ϯ^*p* = 0.009). **g** Increased expression of ZFAS1 in colon cancer compared to unpaired colon epithelium in the GEO GSE103512 array dataset (*n* = 70 Affymetrix array expression, units = normalized Affymetrix array) (*p < 0.001). **h** There is increased ZFAS1 expression with copy number gain and copy number amplification compared to diploid in the RNAseq TCGA data set (*n* = 388) (*p < 0.001, ^Ϯ^*p* < 0.009). **i** There is no difference in the overall clinical patient survival between high and low ZFAS1 expression in the RNAseq TCGA data set (*n* = 192) (RNA-seq expression data dichotomized on basis of median expression) (*p* = 0.11). All RTqPCR experiments were run in duplicate.

Previous studies have shown that the tumor microenvironment can skew data analysis by “contaminating” gene expression in macrodissected tumor samples^10,11^. Therefore, laser capture microdissection was used to investigate lncRNA expression in 23 patients with paired colon adenocarcinoma and normal colon epithelium samples (Table 1, Fig. 1a). Three of the seven lncRNAs (PVT1, GAS5, and ZFAS1) were significantly upregulated and two (FAM83H-AS1 and UCA1) were significantly downregulated in colon adenocarcinoma compared to paired normal colon epithelium samples (Fig. 1b). ZFAS1 was the most significantly increased lncRNA in our validated data set and was therefore selected for further investigation (Fig. 1c). ZFAS1 was significantly increased in expression in three separate sequencing data sets compared to normal tissue (all *p* < 0.05) (Fig. 1d–f). As expected, increased ZFAS1 expression was associated with both copy number gain and amplification (Fig. 1g). There was, however, no difference in the overall survival of the TCGA clinical cohort between high and low ZFAS1 expression (Fig. 1h).

**Table 1:** Clinical details of patients from the University of Louisville Biorepository.

Variable	*N* = 23 *N* (%)
Age at diagnosis (years) (median, interquartile range)	70 (61–77)
Gender	
Male	16 (70)
Female	7 (30)
Race	
Caucasian	17 (74)
African-American	6 (26)
AJCC tumor stage	
Stage I	7 (30)
Stage II	6 (26)
Stage III	5 (22)
Stage IV	5 (22)

### ZFAS1 knockdown is associated with a less aggressive phenotype

**Fig. 2:**
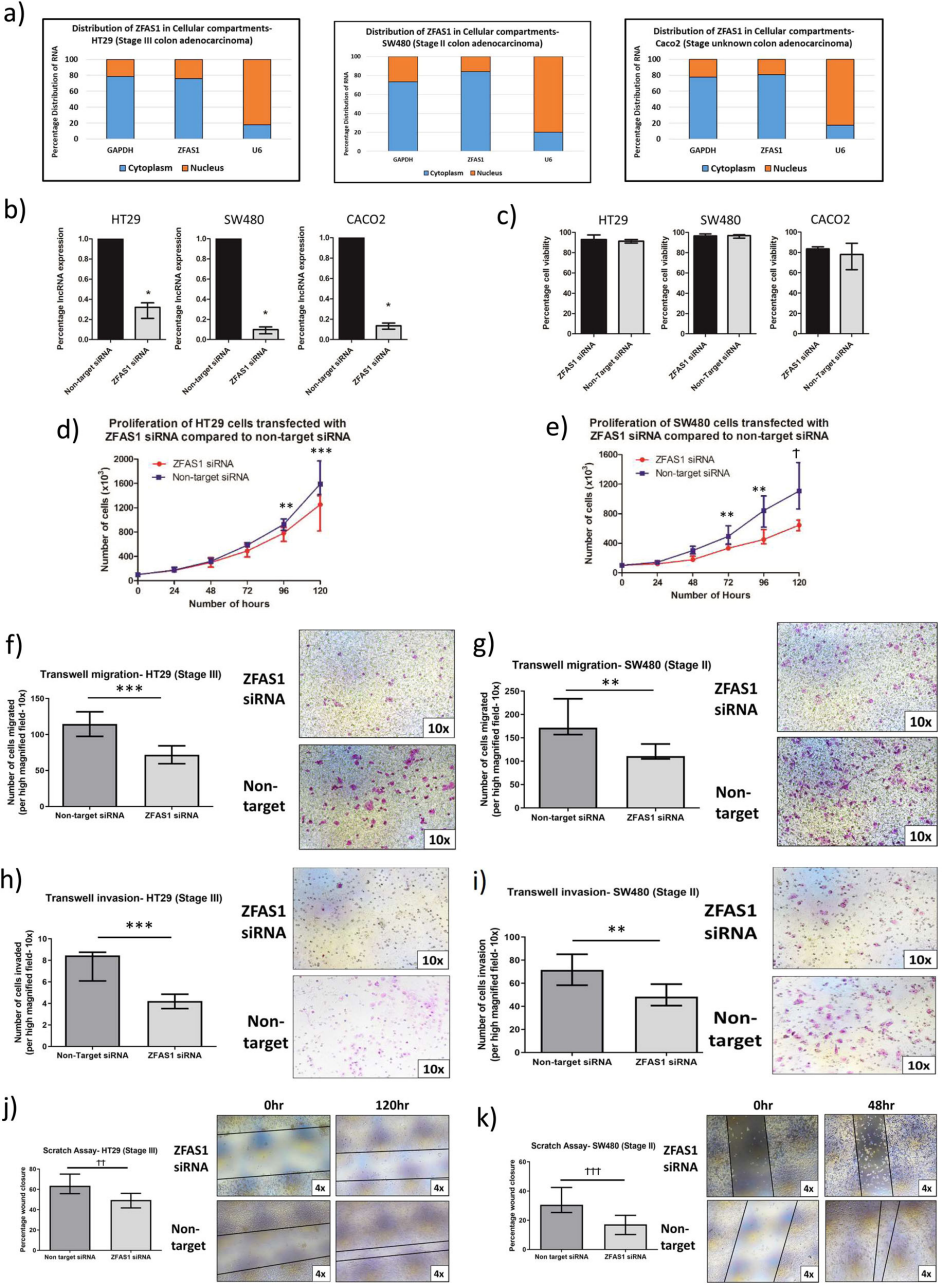
Characterization of ZFAS1 function in vitro. **a** ZFAS1 is principally located in the cytoplasm, compared to the nucleus, measured by RTqPCR in all three colon cancer cell lines (HT29: 76% vs. 24%, *p* = 0.02, SW480: 84% vs. 16%, *p* = 0.03, Caco2: 77% vs. 23%, *p* = 0.005, *n* = 6 replicates). **b** RTqPCR shows efficient knockdown of ZFAS1 with ZFAS1 siRNA in all three cell lines compared to non-target siRNA (all > 70%, all *p < 0.05, *n* = 6 replicates). **c** There is no decrease in the cell viability (percentage of viable cells compared to all counted cells) with ZFAS1 knockdown compared to cells transfected with non-target siRNA (*n* = 6 replicates). **d** There is significant decrease in the cellular proliferation with ZFAS1 knockdown in the HT29 cell lines, by examining the doubling time (34.3 h vs. 30.6 h, *p* = 0.046), the individual counts at 96 h (**p = 0.03), and the counts at 120 h (***p = 0.02) (*n* = 6 replicates). **e** There is significant decrease in the cellular proliferation with ZFAS1 knockdown in the SW480 cell lines, by examining the doubling time (42.5 h vs. 31.5 h, *p* = 0.01), and the individual counts at 72 h (**p = 0.03), 96 h (**p = 0.03), and 120 h (^Ϯ^*p* = 0.01) (*n* = 6 replicates). **f** There is significantly decreased migration of cells with ZFAS1 knockdown in the HT29 cell line (***p = 0.02, *n* = 6 replicates) The panels on the right show representative ×10 magnification fields of the HT29 cell line transwell migration assay (top panel ZFAS1 knockdown, bottom panel non-target siRNA). **g** There is significantly decreased migration of cells with ZFAS1 knockdown in the SW480 cell line (**p = 0.03, *n* = 6 replicates). The panels on the right show representative ×10 magnification fields of the SW480 cell line transwell migration assay (top panel ZFAS1 knockdown, bottom panel non-target siRNA). **h** There is significantly decreased invasion of cells with ZFAS1 knockdown in the HT29 cell line (***p = 0.02, *n* = 6 replicates) The panels on the right show representative ×10 magnification fields of the HT29 cell line transwell invasion (top panel ZFAS1 knockdown, bottom panel non-target siRNA). **i** There is significantly decreased invasion of cells with ZFAS1 knockdown in the SW480 cell line (**p = 0.03, *n* = 6 replicates). The panels on the right show representative ×10 magnification fields of the SW480 cell line transwell invasion (top panel ZFAS1 knockdown, bottom panel non-target siRNA). **j** There was a significantly slower scratch closure of the HT29 cell line with ZFAS1 knockdown compared to control (^ϮϮ^*p* = 0.004, *n* = 11 replicates). The panels on the right show representative ×4 magnification fields of the HT29 scratch assay (top panels scratch closure following ZFAS1 knockdown at time 0 and at 120 h, bottom panels scratch closure with non-target siRNA at time 0 and at 120 h). **k** There was a signficiantly slower scratch closure of the SW480 cell lines with ZFAS1 knockdown compared to control (^ϮϮϮ^*p* < 0.001, *n* = 6 replicates). The panels on the right show representative ×4 magnification fields of the SW480 scratch assay (top panels scratch closure following ZFAS1 knockdown at time 0 and at 48 h, bottom panels scratch closure with non-target siRNA at time 0 and at 48 h).

ZFAS1 was principally located in the cellular cytoplasm of all three cell lines (Fig. 2a). There was robust ZFAS1 knockdown in all three cell lines (all > 70% and *p* < 0.05), without a decrease in cellular viability compared to non-target siRNA (all *p* > 0.05) (Fig. 2b, c). ZFAS1 knockdown was associated with decreased cellular proliferation (Fig. 2d, e) in the HT29 and SW480 cell lines. There was, however, no difference in Caco2 cellular proliferation comparing ZFAS1 knockdown to non-target siRNA, which is likely due to the inherent slow doubling time of the Caco2 cell line^12^. Therefore, for further functional experiments, we focused on the HT29 (epithelial-like) and the SW480 cell line (mesenchymal-like). As such, ZFAS1 knockdown was associated with decreased transwell migration and invasion (Fig. 2f–i) and a slower scratch assay closure (Fig. 2j, k) in the HT29 and SW480 cell lines. As expected, both transwell assays and scratch closure demonstrated slower migration of the HT29 cell line compared to the SW480 cell line. This is likely due to the HT29 cell line having an epithelial-like Consensus Molecular Subtype 3 (CMS3) compared to the mesenchymal-like CMS4 of the SW480 cell line^12^.

### Investigation of experimentally validated miRNA targets of ZFAS1

**Fig. 3:**
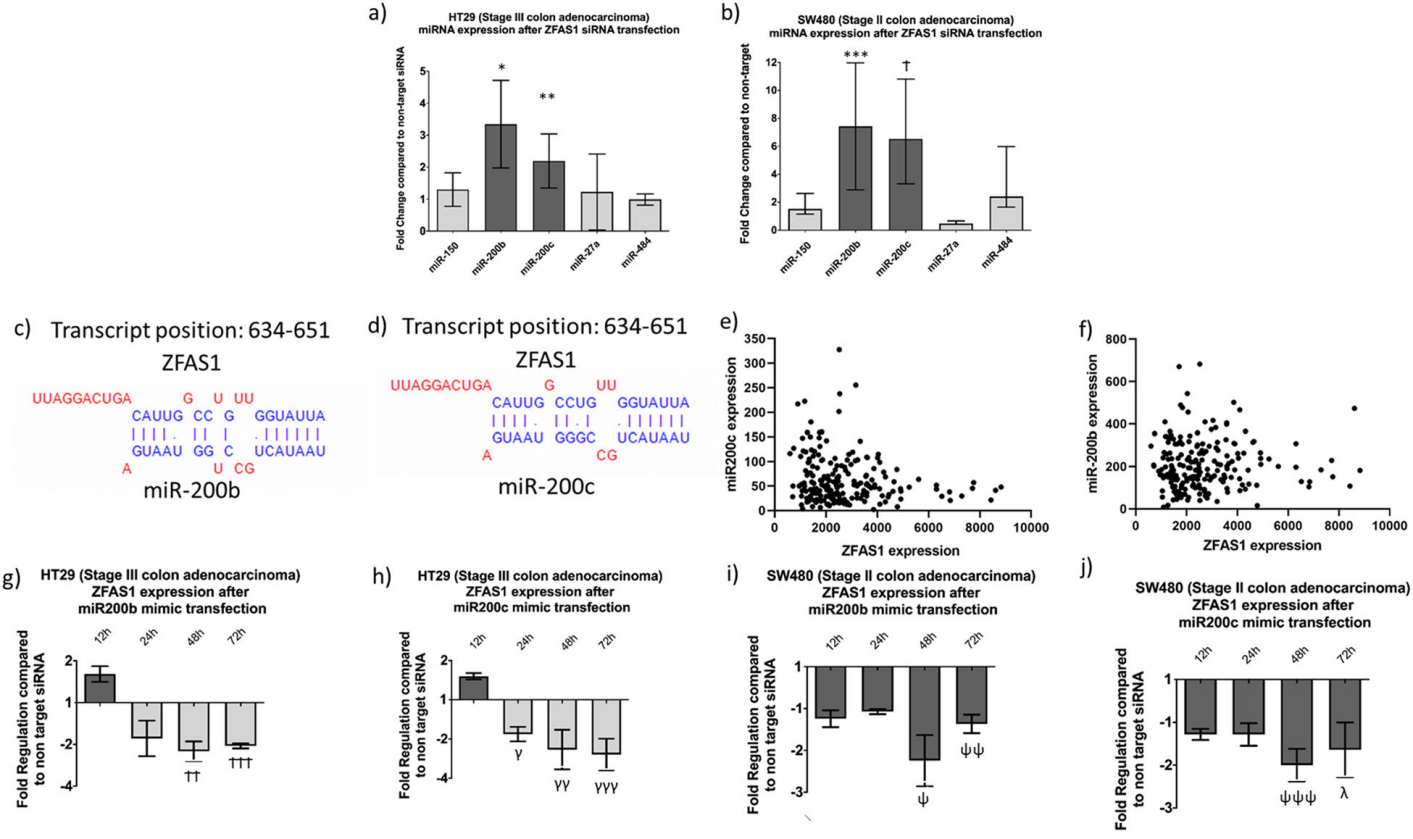
Examination of the interactions between ZFAS1 and target miRNAs. **a** ZFAS1 knockdown leads to increased miR-200b (FC = 3.34, *p = 0.01) and miR-200c (FC = 2.28, **p = 0.012) expression using RTqPCR in the HT29 cell line (*n* = 6 replicates). **b** ZFAS1 knockdown leads to increased miR-200b (FC = 6.69, ***p = 0.005) and miR-200c (FC = 6.53, ^Ϯ^*p* = 0.022) expression using RTqPCR in the SW480 cell line (*n* = 6 replicates)**. c** The experimentally validated site of interaction between ZFAS1 and miR-200b is shown. **d** The experimentally validated site of interaction between ZFAS1 and miR-200c is shown. **e** Using the Cancer Genome Atlas data set, miR200c was inversely correlated with ZFAS1 expression (*n* = 199, RNA-seq and miRNA-seq expression data) (Spearman *r* = 0.120, *p* = 0.09. **f** Using the Cancer Genome Atlas, miR200b was not inversely correlated with ZFAS1 expression (*n* = 199, RNA-seq and miRNA-seq expression data) (Spearman *r* = 0.03, *p* = 0.65). **g** Following transfection with miR-200b mimics, there is signficantly decreased ZFAS1 expression using RTqPCR at 48 (FR = −2.33, ^ϮϮ^*p* = 0.001) and 72 (FR = −2.074, ^ϮϮϮ^*p* = 0.008) hours after transfection (*n* = 6 replicates). **h** Following transfection with miR-200c mimics, there is signficantly decreased ZFAS1 expression using RTqPCR at 24 (FR = −1.74, γ *p* = 0.041), 48 (FR = −2.53, γγ *p* = 0.015) and 72 (FR = −2.78, γγγ *p* = 0.026) hours after transfection in the HT29 cell line (*n* = 6 replicates). **i** Following transfection with miR-200b mimics, there is signficantly decreased ZFAS1 expression using RTqPCR at 48 (FR = −2.33, ^ѱ^*p* = 0.033) and 72 (FR = −1.46, ^ѱѱ^*p* = 0.02) hours after transfectionin the SW480 cell line (*n* = 6 replicates)**. j** Following transfection with miR-200c mimics, there is significanlty decreased ZFAS1 using RTqPCR at 48 (FR = −1.99, ^ѱѱѱ^*p* = 0.03) and 72 (FR = −1.64, λ *p* = 0.02) in the SW480 cell line (*n* = 6 replicates).

As previously mentioned, there are experimentally validated lncRNA-miRNA interactions for ZFAS1 (Table 2) (refs. ^13–20^). Following ZFAS1 knockdown, expression of both miR-200b and miR-200c was significantly increased compared to non-target siRNA (Fig. 3a, b). The lncRNA-miRNA binding sites from the DIANA lncBASE platform are shown in Fig. 3c, d. ZFAS1 was inversely correlated with miR-200c (Spearman *r* = −0.120, *p* = 0.09), but not inversely correlated with miR-200b (Spearman *r* = −0.030, *p* = 0.65) in the TCGA data set (Fig. 3e, f). Although ZFAS1 does not inversely correlate with miR-200b, lncRNA and miRNA expression are dependent on a number of different upstream regulatory molecules^21^. We have previously reported the adverse role of low miR-200b and miR-200c expression with overall survival^22^. Both mir-200b and miR-200c are increased in colorectal cancer compared to paired normal colon epithelium. In addition, both miR-200b and miR-200c are decreased in expression in Stage III/IV compared to Stage I/II colon cancer. Furthermore, transfection with either miR-200b or miR-200c mimics leads to decreased ZFAS1 expression in the both the HT29 and SW480 cell lines (Fig. 3g–j). The direct binding interaction between ZFAS1 and miR-200b between miR-200c has been shown previously through both RNA immunoprecipitation^15^ and an RNA pulldown assay^16^. This defined interaction was examined for subsequent mechanistic and functional assays.

**Table 2:** List of lncRNA-miRNA interactions.

lncRNA	miRNA	Literature citation	Method of experimental verification
ZFAS1	miR-200b	Liu G. et al.^13^	RNA immunoprecipitation
		Zhang F et al.^14^	RNA pulldown assay
	miR-200c	Liu G. et al.^13^	RNA immunoprecipitation
	miR-150	Xia B. et al.^15^	Luciferase reporter assay
		Li T. et al.^16^	RNA pulldown assay
		Wu T. et al.^17^	RNA pulldown assay
		Chen X et al.^18^	RNA pulldown assay
	miR-484	Xie S et al.^19^	RNA immunoprecipitation
	miR-27a	Ye Y et al.^20^	RNA pulldown assay
			Luciferase reporter assay

### ZFAS1 knockdown is associated with changes in the ZEB1 and is mediated through miR-200b and miR-200c signaling

**Fig. 4:**
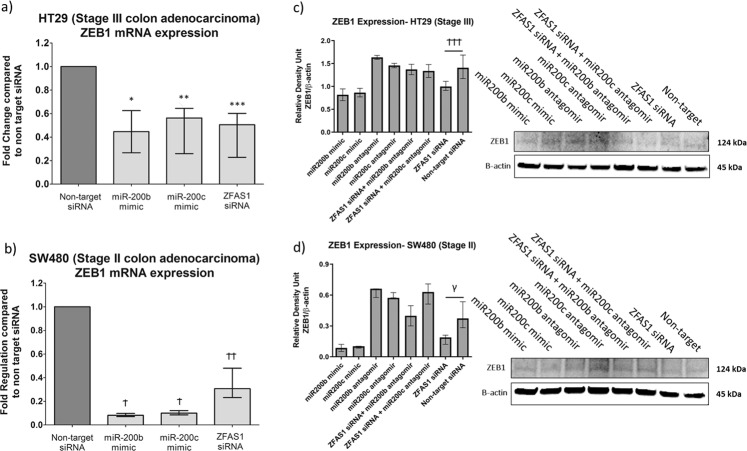
ZFAS1 knockdown is associated with changes in ZEB1 expression. This effect is reversed by dual knockdown of ZFAS1 and miR200b or with ZFAS1 and miR200c. **a** In the HT29 cell line, ZFAS1 knockdown (FR = −2.68, *p = 0.031), miR-200b mimics (FR = −3.19, **p = 0.03), or miR-200c mimics (FR = −1.82, ***p = 0.04) leads to decreased ZEB1 mRNA expression using RTqPCR (*n* = 6 replicates). **b** In the SW480 cell line, ZFAS1 knockdwon (FR = −3.37, ^Ϯ^*p* = 0.034), miR-200b mimics (FR = −12.24, ^Ϯ^*p* < 0.001), or miR-200c mimics (FR = −10.1, ^ϮϮ^*p* < 0.001) knockdown leads to decreased ZEB1 mRNA expression using RTqPCR (*n* = 6 replicates). **c** There is significantly decreased ZEB1 protein expression with ZFAS1 knockdown (^ϮϮϮ^*p* = 0.046) in the HT29 cell line by Western blotting. This effect was reversed by dual knockdown of either ZFAS1 and miR200b or ZFAS1 and miR200c (both *p* > 0.05). As expected, miR200b and miR200c antagomirs increased ZEB1 expression, with reciprocal decreased in ZEB1 expression with miR-200b and miR200c mimics (*n* = 5 replicates). **d** Similarly, there is significantly decreased ZEB1 protein with ZFAS1 knockdown in the SW480 cell line by Western blotting (γ *p* = 0.047). This effect was reversed by dual knockdown of either ZFAS1 and miR200b or ZFAS1 and miR200c (both *p* > 0.05). As expected, miR200b and miR200c antagomirs increased ZEB1 expression, with reciprocal decreased in ZEB1 expression with miR-200b and miR200c mimics (*n* = 5 replicates).

Previous studies have demonstrated the major role of the miR-200 family in cancer signaling, particularly in EMT regulation^4^. Transfection with either ZFAS1 siRNA, miR-200b, or miR-200c mimics leads to ZEB1 mRNA knockdown in both the HT29 and SW480 cell lines (Fig. 4a, b). ZFAS1 knockdown, in turn, leads to decreased ZEB1 protein expression in both cell lines (Fig. 4c, d). Furthermore, dual knockdown of ZFAS1 and miR-200b or ZFAS1 and miR-200c reversed this effect on ZEB1 expression in both the HT29 and SW480 cell lines. As miR-200b and miR-200c act directly on ZEB1, pronounced effects are seen on ZEB1 expression with miRNA mimics and antagomirs.

### Increased ZFAS1 expression and decreased miR-200b or miR-200c expression is associated with adverse clinical outcomes, and ZFAS1 mediates its effect on the E-cadherin vimentin signaling through miR-200b and miR-200c signaling

**Fig. 5:**
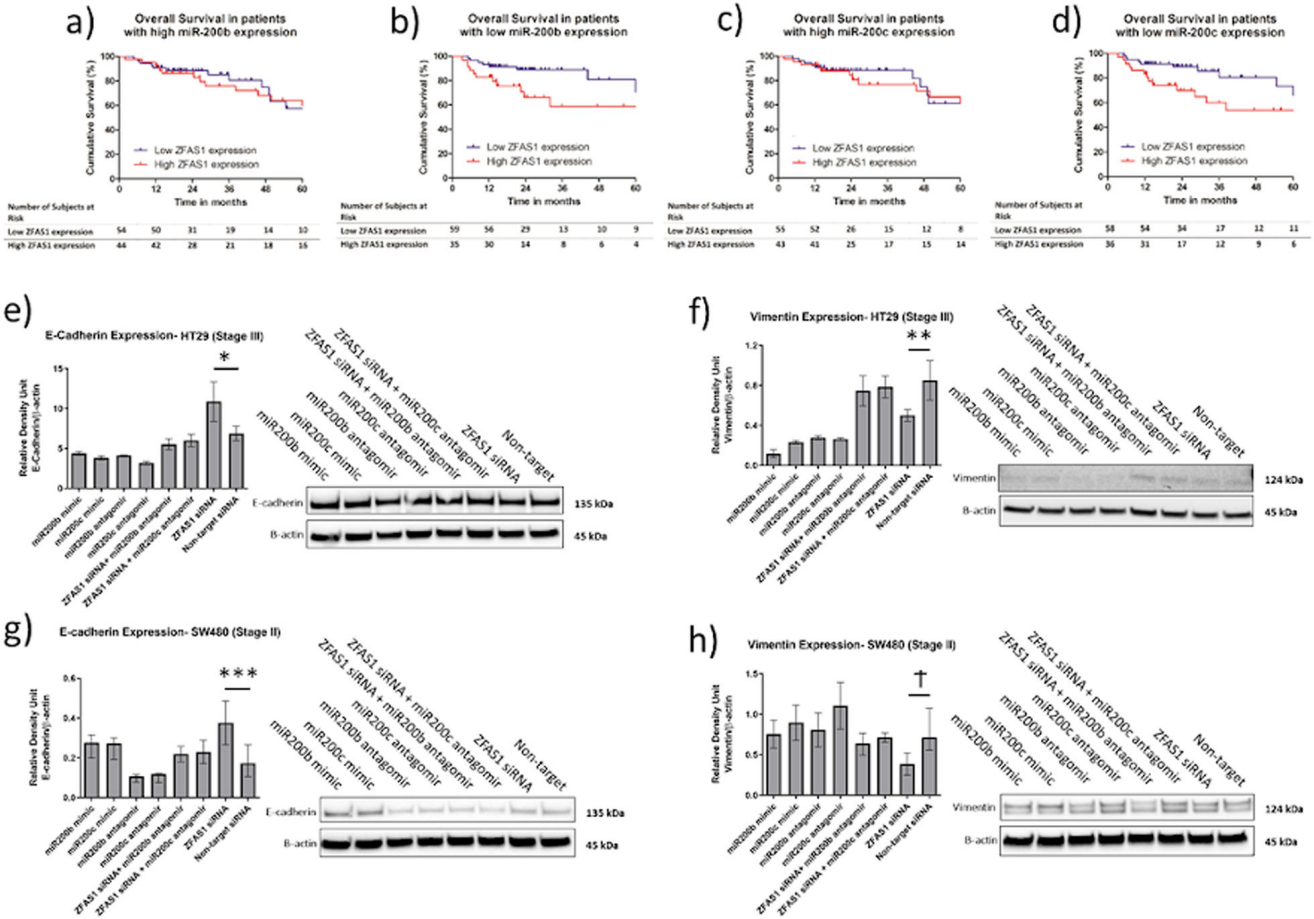
Colon cancer patients with high ZFAS1 and low miR-200b or miR-200c expression have worse overall survival. The effect of ZFAS1 knockdown on E-cadherin and vimentin expresison is reveresed by dual knockdown of ZFAS1 and miR200b or with ZFAS1 and miR200c. **a** Using the TCGA RNAseq and miRNAseq expression data (*n* = 199), there is no difference in the overall survival of colon cancer patients with high miR-200b expression (stratified by median expression) when stratified by ZFAS1 median expression (*p* = 0.95), (**b**) but there is a significant difference in the overall survivial of colon cancer patients with low miR-200b expression when stratified by ZFAS1 expression (*p* = 0.018). **c** There is no difference in the overall survival of colon cancer patients with high miR-200c expression (stratified by median expression) when stratified by ZFAS1 expression (*p* = 0.81), (**d**) but there is a significant difference in the overall survivial of colon cancer patients with low miR-200c expression when stratified by ZFAS1 median expression (*p* = 0.048). **e** There is significantly increased E-cadherin protein with ZFAS1 knockdown (*p = 0.022) in the HT29 cell line by Western blotting. This effect was reversed by dual knockdown of either ZFAS1 and miR200b or ZFAS1 and miR200c (both *p* > 0.05). As expected, transfection with miR200b and miR200c antagomirs reduces E-cadherin expression (both *p* < 0.05). Transfection with both miR200b and miR200c mimics did not lead to the expected increase in E-cadherin expression (*n* = 5 replicates). **f** There is significantly decreased vimentin protein with ZFAS1 knockdown (**p = 0.025) in the HT29 cell line by Western blotting. This effect was reversed by dual knockdown of either ZFAS1 and miR200b or ZFAS1 and miR200c (both *p* > 0.05). Unexpectedly, miR200b and miR200c antagomirs did not lead to increased vimentin expression. As expected, transfection with miR200b and miR200c mimics reduced vimentin expression (both *p* < 0.05) (*n* = 5 replicates). **g** There is significantly increased E-cadherin protein with ZFAS1 knockdown (***p = 0.021) in the SW480 cell line by Western blotting. This effect was reversed by dual knockdown of either ZFAS1 and miR200b or ZFAS1 and miR200c (both *p* > 0.05). As expected, transfection with miR200b and miR200c antagomirs reduces E-cadherin expression (both *p* < 0.05). Transfection with miR200b and miR200c mimics leads to increased E-cadherin expression (*n* = 5 replicates). **h** There is significantly decreased vimentin protein with ZFAS1 knockdown (^Ϯ^*p* = 0.036) in the HT29 cell line by Western blotting. This effect was reversed by dual knockdown of either ZFAS1 and miR200b or ZFAS1 and miR200c (both *p* > 0.05). miR200b and miR200c antagomirs led to increased vimentin expression, but this was not statistically significant. Transfection with miR200b and miR200c mimics did not lead to decreased vimentin expression (*n* = 5 replicates).

As previously mentioned, ZFAS1 expression alone is not associated with adverse clinical outcomes. However, when ZFAS1 expression is stratified by both miR-200b (Fig. 5a, b) and miR-200c expression (Fig. 5c, d), high ZFAS1 expression is associated with worse overall survival in the setting of low miR-200b (*p* = 0.018) and low miR-200c (*p* = 0.048) expression. This suggests that patients with high ZFAS1 expression and low miR-200b or low miR-200c expression have a particularly poor genomic signature. As hypothesized, downstream of ZEB1, ZFAS1 knockdown leads to increased E-cadherin expression as well as decreased vimentin protein expression (Fig. 5e–h) in both the HT29 and SW480 cell lines. This regulatory effect on E-cadherin and vimentin expression was attenuated with dual transfection of ZFAS1 and miR-200b or ZFAS and miR-200c in both the HT29 and SW480 cell lines. Interestingly, we did not observe an increase in the E-cadherin expression with miR-200b or miR-200c mimic transfection. There is low endogenous expression of ZEB1 in the HT29 cell line, and this may explain the inability of miR-200b or miR-200c to indirectly regulat E-cadherin expression. This explanation may also account for the differences seen in vimentin expression in the HT29 cell line.

### miR-200b and miR-200c knockdown partially reverse the functional effect of ZFAS1 knockdown on proliferation, migration, and invasion

**Fig. 6:**
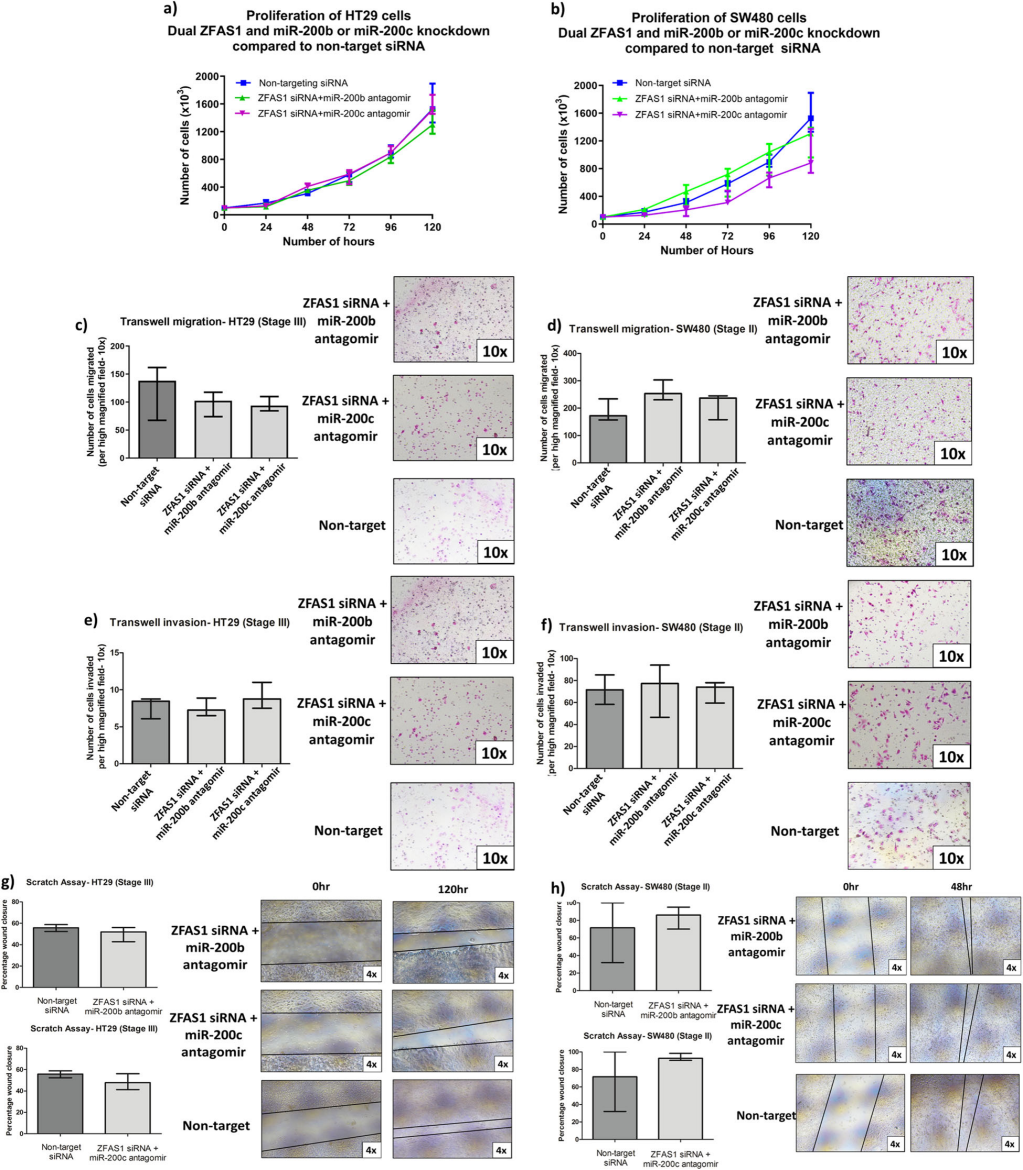
Knockdown of miR-200b or miR-200c partially reverses the effect on proliferation, but reverses the effect on migration and invasion. **a** In the HT29 cell line, there is no difference in cell proliferation following dual ZFAS1 and miR-200b knockdown compared to non-target siRNA, when measured by doubling time (*p* = 0.46), but there is more rapid proliferation with dual knockdown of ZFAS1 and miR-200c compared to non-target siRNA (*doubling time: 26.8 h vs. 30.6 h, *p* = 0.01) (*n* = 6 replicates). **b** In the SW480 cell line, cell proliferation following dual ZFAS1 and miR-200b knockdown is slower than compared to non-target siRNA (**doubling time: 35.9 vs. 30.5, *p* = 0.06), but there was no difference in proliferation with dual knockdown of ZFAS1 and miR-200c compared to non-target siRNA (*p* = 0.55) (*n* = 6 replicates). **c**, **d** In the HT29 and SW480 cell lines, there is no difference in the transwell migration with both ZFAS1 and miR-200b or ZFAS1 andmiR-200c knockdown compared to non-target siRNA (all *p* > 0.05) (*n* = 6 replicates), The panels on the right of each figure show representative ×10 magnification fields of the HT29 and SW480 cell lines transwell migration assay (top panel ZFAS1 knockdown + miR-200b antagomir, middle panel ZFAS1 knockdown + miR-200c antagomir, bottom panel non-target siRNA). **e**, **f** In the HT29 and SW480 cell lines, there was no difference in the transwell invasion with both ZFAS1 and miR-200b or ZFAS1 and miR-200c knockdown compared to non-target siRNA (all *p* > 0.05) (*n* = 6 replicates). The panels on the right of each figure show representative ×10 magnification fields of the HT29 and SW480 cell lines transwell migration assay (top panel ZFAS1 knockdown + miR-200b antagomir, middle panel ZFAS1 knockdown + miR-200c antagomir, bottom panel non-target siRNA). **g** In the HT29 cell line, there was no difference in scratch closure in both ZFAS1 and miR-200b or ZFAS1 and miR-200c knockdown compared to non-target siRNA (all *p* > 0.05) (*n* = 6 replicates). The panels on the right show representative ×4 magnification fields of the HT29 scratch assay (top panels scratch closure following dual ZFAS1 and miR-200b knockdown at time 0 and at 120 h, middle panels scratch closure following dual ZFAS1 and miR-200c knockdown at time 0 and 120 h, bottom panels scratch closure with non-target siRNA at time 0 and at 120 h). **h** In the SW480 cell line, there was no difference in scratch closure in both ZFAS1 and miR-200b or ZFAS1 and miR-200c knockdown compared to non-target siRNA (all *p* > 0.05) (*n* = 6 replicates). The panels on the right show representative ×4 magnification fields of the SW480 scratch assay (top panels scratch closure following dual ZFAS1 and miR-200c knockdown at time 0 and at 48 h, middle panels scratch closure following dual ZFAS1 and miR-200c knockdown at time 0 and 48 h, bottom panels scratch closure with non-target siRNA at time 0 and at 48 h).

To validate these signaling cascade results, we performed functional cellular assays with dual knockdown of both ZFAS1 and miR-200b or ZFAS1 and miR-200c. In the HT29 cell line, there was no difference in cellular proliferation with dual knockdown of ZFAS1 and miR-200b compared to non-target. However, there was a significant difference in the doubling time of cells with dual knockdown of ZFAS1 and miR-200c compared to non-target (Fig. 6a, Supplementary Table 4). In the SW480 cell line, there was no difference in the doubling time following ZFAS1 and miR-200c knockdown compared to non-target, but there were significant differences in the individual cell counts at 96 h (*p* = 0.003) and 120 h (*p* = 0.02). Following knockdown with ZFAS1 and miR-200b, there was a slower doubling time of cells as compared to non-target (Fig. 6b, Supplementary Table 4). In both cell lines, under both conditions, there was no difference in the transwell migration or invasion compared to non-target (all *p* > 0.05) (Fig. 6c–f). In the HT29 cell line, there was a significant difference in the scratch closure of cells following dual ZFAS1 and miR-200c knockdown compared to non-target (*p* = 0.02), but there was no difference in the dual ZFAS1 and miR-200b knockdown. In addition, there was no difference in the scratch closure in either of the SW480 dual knockdown conditions (Fig. 6g, e).

### ZFAS1 knockdown is associated with decreased tumor growth in vivo

**Fig. 7:**
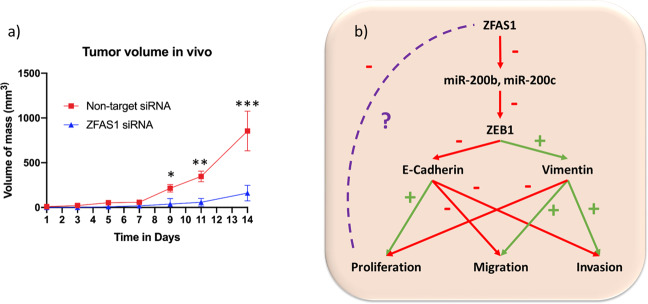
ZFAS1 knockdown is associated with decreased tumor growth in vivo compared to not-target siRNA. **a** ZFAS1 knockdown was associated with decreased tumor volume in a mouse xenograft model compared to non-target siRNA, with significant differenes observed from day 3 after mass was first identified in each mouse. The differences were most pronounced from day 7 up until day 14 when the experiment was terminated (*N* = 4 replicates) (*p = 0.006, **p < 0.001, ***p = 0.024). **b** Illustration of the signaling cascade demonstrated by these results.

Previous experiments describe the clinical relevance and the mechanisms of action of ZFAS1 in colorectal cancer cell lines, but it is important to explore its mechanism of action in vivo. Using a mouse model, ZFAS1 knockdown was associated with decreased tumor volume compared to non-target siRNA (Fig. 7a). A summary of the results of this study are illustrated in Fig. 7b.

## Discussion

We hypothesized that using a RNA-sequencing data set to compare the entire transcriptome of colon adenocarcinoma tissue to paired normal colon epithelium tissue would allow us to investigate differentially expressed lncRNAs that are critical in colorectal carcinogenesis. We validated the expression of ZFAS1, identifying it as a major regulator of tumor progression in CRC, through the critical miR-200/ZEB1/E-cadherin, vimentin signaling cascade.

There are variable results when examining ZFAS1 as a mediator of carcinogenesis in the literature^6^. ZFAS1 has been shown to have a tumor suppressive function in breast cancer by decreasing cellular proliferation and migration^23,24^. In hepatocellular carcinoma, ZFAS1 can have both a tumor suppressive and oncogenic function. For example, it can act as a tumor suppressor by methylating miR-9 in hepatocellular carcinoma^25^, but can also promote cancer metastasis through miR-150 binding^16^. The majority of studies, however, demonstrate that ZFAS1 has an oncogenic function, many indicating that ZFAS1 can regulate both proliferation and migration^13,15^. Recently, *Chen et al*. reported that ZFAS1 could regulate proliferation, migration, and invasion in colorectal cancer^18^. ZFAS1 could mediate an indirect regulation of VEGFA by directly binding miR-150. The results of our experiments support the functional data reported by Chen et al, in that ZFAS1 knockdown leads to decreased proliferation and migration. The direct binding interaction between ZFAS1 and the miR-200 family has been shown previously through different mechanistic assays including RNA immunoprecipitation^15^ and RNA pulldown^16^. We have validated this interaction in both colorectal cancer patients and in colorectal cancer cell lines.

Interestingly, we also investigated the interaction with miR-150, but we did not find a reciprocal expression change. This finding contrasts with Chen et al., as they reported that miR-150 had a direct binding interaction with ZFAS1 in the HCT116 cell line. This explanation for this discrepancy is unclear, however, and requires further investigation. Nevertheless, both sets of data support the concept that lncRNAs can have multiple mechanisms of action through different signaling cascades. This attribute has been described as both a positive and negative aspect regarding their potential utility^26,27^.

Non-coding RNAs are being investigated as a small molecule therapeutic strategy for different diseases. There are a number of clinical trials exploring both miRNA and siRNA technology as a therapeutic strategy^28^. For example, a novel small molecule targeting miR-122 has been effective in reducing RNA levels of hepatitis C virus in a phase 2 clinical trial^29^. In the future, lncRNAs and other non-coding RNAs may have a major role in the therapeutic management of different diseases.

Many studies demonstrate that miRNA can regulate lncRNAs through the competitive endogenous RNA mechanism, but few examine upstream regulators. We report that there is copy number amplification seen with ZFAS1 in the TCGA colon adenocarcinoma cohort. Additionally, transcription factor, SP1, has been shown to transcriptionally increase ZFAS1 in a number of different cancer types^6^. This suggests that modulating specific transcription factors adds a further layer of complexity when studying the relationship between lncRNAs and protein coding genes. This complex regulatory network could partially explain why we do not find a clear inverse relationship between ZFAS1 and miR200b or miR200c, as a number of different upstream regulators can affect their expression.

The results of these experiments highlight areas requiring further investigation. There is some conflict between the results from the TCGA differential gene expression and our results from laser capture microdissection. The tissue that is used for RNA-seq in the TCGA data is from a macrodissected tumor sample. Therefore, it includes not only cancer cells, but also other cells from the tumor microenvironment^10^. Previous studies have shown that molecules, such as miRNA, can have different expression, depending on the location from which the RNA is extracted from a cancer specimen (e.g., superficial tumor, deep border etc)^11,30^. In addition, cells from within the tumor microenvironment may express or secrete lncRNAs in order to induce an effect on cancer cells^31^. In this case, whole tissue dissection may lead to the false conclusion that the lncRNA is expressed from the cancer cells instead of from cells within the tumor microenvironment. TGF-β is a major inducing agent of EMT^32^, and recent studies have demonstrated that it can be secreted by macrophages and other immune cells in the tumor microenvironment^33^. This could be investigated through the use of transwell chambers or by treating colon adenocarcinoma cell lines with cultured macrophage secretions, as performed by Jahangiri et al.^31^. Although our study demonstrates the role of ZFAS1 signaling through regulation of miR-200b and miR-200c, ZFAS1 interacts with a number of other miRNAs, suggesting further work is required to fully delineate the function of ZFAS1 and other lncRNAs.

There are a number of limitations to this work. As previously mentioned, lncRNAs can mediate an effect on a number of different signaling pathways; however, we focused on a single signaling pathway to help delineate the mechanism of action of ZFAS1. There are five members of the miR-200 family, and we restricted our study to two of these, miR-200b and miR-200c, since they have been experimentally verified to interact with ZFAS1. Although miR-200b, miR-200c, and miR-429 are part of the same functional cluster, we did not examine the role of miR-429, as it was not an experimentally validated target of ZFAS1. Further studies could investigate the role of other members of the miR-200 family in relation to ZFAS1. Although we selected lncRNAs that are increased in expression in colon cancer compared to normal colon epithelium, there are a large number of lncRNAs that are downregulated in expression. Due to the large size of lncRNA, stable transfection is challenging, but some investigators have suggested the use of CRISPR-activation technology to stably increase the expression of an lncRNA in vitro^34,35^. Further experiments examining miRNA, gene expression, and proliferations markers in the xenograft tumors is warranted.

In conclusion, we identified and validated that ZFAS1 can regulate EMT through a reciprocal interaction with both miR-200b and miR-200c. Knockdown of ZFAS1 can lead to a less aggressive phenotype in colon cancer cell lines, reduction in the mesenchymal marker vimentin, and an increase in the epithelial marker E-cadherin. This indicates that ZFAS1 is a major regulator of cancer aggressiveness and could be a target for therapeutic intervention in the future.

## Methods

A detailed methods section is described in the Supplementary Methods

### Ethics statement

This study was approved by the institutional review board of the University of Louisville and the Institutional Animal Care and Use Committee of the University of Louisville.

### The Cancer Genome Atlas and Gene Expression Omnibus (GEO)

The raw sequencing data for colon adenocarcinoma patients were identified from the Genomic Data Commons portal (https://portal.gdc.cancer.gov/). An R package “edgeR” was used to identify differentially expressed genes between a 40 patient subset that had both colon adenocarcinoma and normal colon epithelium RNA-seq data files^36,37^. Heatmap was generated using MORPHEUS (https://software.broadinstitute.org/morpheus).

Two paired data sets (colon cancer tissue and normal colon mucosa from the same patient) GSE104836 (*N* = 10) and GSE95132 (*N* = 10), and one unpaired data set (colon cancer tissue and normal colon mucosa from different patients), GSE103512 (*N* = 70), were downloaded from the GEO database.

### Bioinformatics analysis

Each lncRNA was entered to the bioinformatics prediction program DIANA-lncBASE v2.0 (ref. ^9^). lncRNA targets and signaling pathways were identified using Ingenuity Pathway Analysis (Qiagen, Hilden Germany.)

### Patient samples and laser capture microdissection

Fresh frozen samples of paired colon adenocarcinoma and normal adjacent colon epithelium from 23 patients were obtained from the University of Louisville Surgical Biorepository. Tissue sections were mounted on negatively-charged slides and stained using the Applied Biosystems Arcturus Staining Kit (Thermo Fisher Scientific, Bedford, MA). The ArcturusXT^TM^ Laser Capture Microdissection System was used to isolate the tissue of interest. RNA was extracted using the PicoPure^TM^ RNA Isolation Kit (Thermo Fisher Scientific, Waltham, MA).

### Cell lines

The HT29 (ATCC^®^ HTB-38^TM^), SW480 (ATCC^®^ CCL-228^TM^), and Caco2 (ATCC^®^ HTB-37^TM^) colon adenocarcinoma cell lines were purchased new from the American Type Culture Collection (Manassas, VA). Cell lines were authenticated using Short Tandem Repeat profiling (ATCC, Manassas VA) and tested for mycoplasma using the MycoFluor^TM^ Mycoplasma Detection Kit (Life Technologies, Carlsbad, CA).

### RNA interference

Small interfering RNA (siRNA) (Dharmacon^TM^, Lafayette, CO), miRNA mimics (Dharmacon^TM^) and miRNA antagomirs (Life Technologies) were purchased (Supplementary Table 1). All transfections were performed using Dharmafect-1 transfection reagent (Dharmacon^TM^).

### RNA extraction

The Protein and RNA Isolation System^TM^ (Life Technologies^®^) was used to extract nuclear and cytoplasmic compartment RNA. Total RNA extracted using the Qiagen miRNeasy Mini kit (Qiagen). RNA concentration and purity were assessed using Nanodrop^®^ 2000 spectrophotometry (Thermo Fisher Scientific).

### Real-time quantitative polymerase chain reaction (RT-qPCR)- mRNA and lncRNA quantification

Complementary DNA (cDNA) was generated using the Superscript^TM^ IV VILO^TM^ Master Mix (Life Technologies). qPCR was performed using TaqMan Fast Advanced Master Mix (Life Technologies) and specific TaqMan^®^ probes (Supplementary Table 2). The gene expression levels were normalized to GAPDH.

### miRNA quantification

cDNA was generated from RNA samples using the TaqMan^®^ miRNA reverse transcription kit (Life Technologies). qPCR was performed using cDNA and specific TaqMan^®^ miRNA probes (Supplementary Table 2). The miRNA levels were normalized to U6.

All reactions were performed on a Step-One Plus RT-qPCR system (Life Technologies), and a cycle threshold (Ct) of 0.1 was used to calculate ΔCt values for analysis using the comparative ΔCt method^38^.

### Western blotting

Cells were lysed using radio immune-precipitation assay buffer and protein concentration determined using the bicinchoninic acid assay. The primary antibodies are listed in Supplementary Table 3. Cell lysates were separated by NuPAGE^®^ MOPS SDS, transferred to a nitrocellulose membrane, and developed using a ChemiDoc MP imager (BioRad, Hercules, CA).

### Cell proliferation and viability

HT29, SW480, and Caco2 cells were plated at a concentration of 1 × 10^5^ cells/well in 2% Fetal Bovine Serum (FBS) supplemented media. The media was changed to 10% FBS supplemented media after 24 h. Daily cell counts and percentage of viable cells, using Trypan blue, were obtained every 24 h from 0 to 120 h using an automatic cell counter (TC20^TM^ Bio-Rad Laboratories, Hercules, CA).

### Scratch assay

HT29 and SW480 cells were plated at 1 × 10^6^ cells/well in a 12-well plate. A scratch was made using a sterile 20 μl pipette tip. Photos (Nikon Eclipse TS100) were taken every 24 h from 0 to 120 h or until complete scratch closure (×4 magnification). The percentage scratch closure for each time point was compared to 0 h.

### Transwell migration and invasion

SW480 cells were seeded at 2 × 10^5^ cells/well, and HT29 cells were seeded at 5 × 10^5^ cells/well into a transwell migration (COSTAR, 8.0 μm pore polycarbonate membrane) or invasion insert (COSTAR, 8.0 μm pore Corning BioCoat Matrigel Invasion Chambers), as per the manufacturer’s instructions. Complete media (supplemented with 10% FBS) was the chemoattractant, and plates were incubated at 37 °C for 24 h. Each insert was stained with the Diff-Quik staining kit (Electron Microscopy Sciences, Hatfield, PA), and six representative images were taken (×10 magnification). Migrated cells in each field were manually counted.

### In vivo tumor growth

Six-week old male Nu/J mice were purchased from the Jackson Laboratory (Bar Harbor, Maine). Each mouse was injected with 5 × 10^6^ cells in 200 µL PBS. Mass volume was calculated using the formula: Volume = 0.5 × longitude diameter × (latitudinal diameter)^2^, every 3 days up to 14 days post mass identification.

### Statistical analysis

All reactions were performed in duplicate, with the average being used for analysis. Data are presented as median (interquartile range). The chi-squared test, or Fischer’s exact test, were used to compare categorical variables. The Mann–Whitney U test and Wilcoxon signed-rank test were used to compare continuous variables. Differences in overall survival were compared with the Log-rank test. Statistical analysis was performed using SPSS v26.0 (IBM Corp, Armonk, NY). Graphs were created using GraphPad Prism v6.01 (Graphpad Software Inc. La Jolla, CA). Statistical significance was defined as *p* < 0.05.

## Supplementary information

Supplementary Figure Legends

Supplementary Table 1

Supplementary Table 2

Supplementary Table 3

Supplementary Table 4

Supplementary Figure 1

Supplementary Figure 2

Supplementary methods

## Data Availability

Available upon request
