# Supplementary material for: Long non-coding RNA ZFAS1 is a major regulator of epithelial-mesenchymal transition through miR-200/ZEB1/E-cadherin, vimentin signaling in colon adenocarcinoma
Source: Cell Death Discov. 2021 Mar 26;7:61. doi: 10.1038/s41420-021-00427-x (PMC7998025; doi:10.1038/s41420-021-00427-x)
Supplement: Supplementary file 1 — Supplementary Figure Legends [file 41420_2021_427_MOESM1_ESM.docx]

**Supplementary Table 1. List of primers used for RT-qPCR.**

**Supplementary Table 2. List of products used for RNA interference.**

**Supplementary Table 3. List of antibodies used for western blotting.**

**Supplementary Table 4. Differences in the doubling time of the HT29 and SW480 cell lines, when comparing dual knockdown of ZFAS1 + miR-200b antagomir to non-target siRNA, and comparing dual knockdown of ZFAS1 + miR-200c antagomir to non-target siRNA.**

**Supplementary Figure 1. Pipeline for selection of lncRNA for study**

**Supplementary Figure 2. Uncropped Representative Western Blots**

1. Figure 4c. Uncropped western blot for ZEB1 (124kDa) in the HT29 cell line. The upper red box corresponds to the ZEB1 protein and the lower red box corresponds to b-actin (45kDa). Each of the experimental conditions are labelled above the blot.
2. Figure 4d. Uncropped western blot for ZEB1 (124kDa) in the SW480 cell line. The left blot represents the exposure taken for ZEB1, with the red box corresponding to the ZEB1 protein. The right blot represents the earlier exposure taken for b-actin with the red box corresponding to b-actin (45kDa). Each of the experimental conditions are labelled above the blot.
3. Figure 5e. Uncropped western blot for E-cadherin (135kDa) in the HT29 cell line. The left blot represents the exposure taken for E-cadherin, with the red box corresponding to the E-cadherin protein. The right blot represents the later exposure taken for b-actin with the red box corresponding to b-actin (45kDa). Each of the experimental conditions are labelled above the blot.
4. Figure 5f. Uncropped western blot for Vimentin (57kDa) in the HT29 cell line. The left blot represents the exposure taken for Vimentin, with the red box corresponding to the Vimentin protein. The right blot represents the earlier exposure taken for b-actin with the red box corresponding to b-actin (45kDa). Each of the experimental conditions are labelled above the blot.
5. Figure 5g. Uncropped western blot for E-cadherin (135kDa) in the SW480 cell line. The upper red box corresponds to the E-cadherin protein and the lower red box corresponds to b-actin (45kDa). Each of the experimental conditions are labelled above the blot.
6. Figure 5h. Uncropped western blot for Vimentin (57kDa) in the SW480 cell line. The left blot represents the exposure taken for Vimentin, with the red box corresponding to the Vimentin protein. The right blot represents the earlier exposure taken for b-actin with the red box corresponding to b-actin (45kDa). Each of the experimental conditions are labelled above the blot.
