# Supplementary material for: Long non-coding RNA ZFAS1 is a major regulator of epithelial-mesenchymal transition through miR-200/ZEB1/E-cadherin, vimentin signaling in colon adenocarcinoma
Source: Cell Death Discov. 2021 Mar 26;7:61. doi: 10.1038/s41420-021-00427-x (PMC7998025; doi:10.1038/s41420-021-00427-x)
Supplement: Supplementary file 2 — Supplementary Table 1 [file 41420_2021_427_MOESM2_ESM.docx]

Supplementary Table 1. List of primers used for RT-qPCR.

| Gene Name | Ensembl ID/miR Base ID | Company | Catalogue No. |
| --- | --- | --- | --- |
| ZFAS1 | ENSG00000177410 | ThermoFisher | Hs01379985 M1 |
| H19 | ENSG00000130600 | ThermoFisher | Hs00399294_g1 |
| GAS5 | ENSG00000234741 | ThermoFisher | Hs03464472_m1 |
| PVT1 | ENSG00000249859 | ThermoFisher | Hs00413039_m1 |
| FAM83H-AS1 | ENSG00000282685 | ThermoFisher | Hs01064424_S1 |
| FER1L4 | ENSG00000088340 | ThermoFisher | Hs00957065_g1 |
| ZEB1 | ENSG00000148516 | ThermoFisher | Hs01566408_m1 |
| GAPDH | ENSG00000111640 | ThermoFisher | Hs02786624_g1 |
| hsa-miR-200b | MI0000342 | ThermoFisher | 002251 |
| hsa-miR-200c | MI0000650 | ThermoFisher | 002300 |
| hsa-miR-150 | MI0000479 | ThermoFisher | 000473 |
| hsa-miR-484 | MI0002468 | ThermoFisher | 001821 |
| hsa-miR-27a | MI0000085 | ThermoFisher | 000408 |
| RNU6 | NR_004394 | ThermoFisher | 001973 |
