# Supplementary material for: Long non-coding RNA ZFAS1 is a major regulator of epithelial-mesenchymal transition through miR-200/ZEB1/E-cadherin, vimentin signaling in colon adenocarcinoma
Source: Cell Death Discov. 2021 Mar 26;7:61. doi: 10.1038/s41420-021-00427-x (PMC7998025; doi:10.1038/s41420-021-00427-x)
Supplement: Supplementary file 3 — Supplementary Table 2 [file 41420_2021_427_MOESM3_ESM.docx]

Supplementary Table 2. List of products used for RNA interference.

| Gene Name | Product | Company | Catalogue No. |
| --- | --- | --- | --- |
| ZFAS1 | siRNA | Dharmacon | R-034485-00-0005 |
| PVT1 | siRNA | Dharmacon | R-029357-00-0005 |
| GAS5 | siRNA | Dharmacon | R-188293-00-0005 |
| Non-target siRNA | siRNA | Dharmacon | D-001320-10-05 |
| miR-200b mimic | microRNA mimic | Dharmacon | C-300582-07-005 |
| miR-200c mimic | microRNA mimic | Dharmacon | C-300646-05-0005 |
| miR-200b antagomir | miR Vana miRNA inhibitor | ThermoFisher | MH11714 |
| miR-200c antagomir | miR Vana miRNA inhibitor | ThermoFisher | MH10492 |
