# Supplementary material for: Long non-coding RNA ZFAS1 is a major regulator of epithelial-mesenchymal transition through miR-200/ZEB1/E-cadherin, vimentin signaling in colon adenocarcinoma
Source: Cell Death Discov. 2021 Mar 26;7:61. doi: 10.1038/s41420-021-00427-x (PMC7998025; doi:10.1038/s41420-021-00427-x)
Supplement: Supplementary file 4 — Supplementary Table 3 [file 41420_2021_427_MOESM4_ESM.docx]

Supplementary Table 3. List of antibodies used for western blotting.

| Gene Name | Company | Catalogue No. | Concentration |
| --- | --- | --- | --- |
| *Primary antibodies* |  |  |  |
| ZEB1 | Santa Cruz Biotechnology | Sc-515797 x | 1:100 |
| E-Cadherin | Cell Signaling Technology | 24E10 | 1:5,000 |
| Vimentin | Cell Signaling Technology | D21H3 | 1:5,000 |
| β-actin | Cell Signaling Technology | 8H10D10 | 1:10,000  1:50,000 |
| *Secondary antibodies* |  |  |  |
| HRP-conjugated anti-rabbit antibody | Cell Signaling Technology | 7076S | 1:5,000 |
| HRP-conjugated anti-mouse antibody | Cell Signaling Technology | 7074S | 1:5,000 |
