# Supplementary material for: Long non-coding RNA ZFAS1 is a major regulator of epithelial-mesenchymal transition through miR-200/ZEB1/E-cadherin, vimentin signaling in colon adenocarcinoma
Source: Cell Death Discov. 2021 Mar 26;7:61. doi: 10.1038/s41420-021-00427-x (PMC7998025; doi:10.1038/s41420-021-00427-x)
Supplement: Supplementary file 5 — Supplementary Table 4 [file 41420_2021_427_MOESM5_ESM.docx]

Supplementary Table 4. Differences in the doubling time of the HT29 and SW480 cell lines, when comparing dual knockdown of ZFAS1 + miR-200b antagomir to non-target siRNA, and comparing dual knockdown of ZFAS1 + miR-200c antagomir to non-target siRNA.

|  | HT29 | | SW480 | |
| --- | --- | --- | --- | --- |
|  | Doubling time | P-value | Doubling time | P-value |
| ZFAS1 siRNA + miR-200b antagomir | 29.1 | 0.46 | 35.9 | 0.06 |
| ZFAS1 siRNA + miR-200c antagomir | 26.8 | 0.02 | 31.0 | 0.55 |
| Non-target siRNA | 30.6 |  | 30.5 |  |

Note: A smaller doubling time = faster proliferation. Doubling time= (T_2_-T_1_)x(LN2/LN(C_2_/C_1_), where T_2_ = time at the end of incubation, T_1_ = time at start of incubation, LN = natural logarithm, C_2_ = Count at the end of incubation, C_1_ = Count at the start of incubation. Comparisons are made between the ZFAS1 siRNA + miR-200b antagomir compared to non-target siRNA, or between the ZFAS1 siRNA + miR-200c antagomir compared to non-target siRNA conditions.
