# Supplementary figures and images for: Long non-coding RNA ZFAS1 is a major regulator of epithelial-mesenchymal transition through miR-200/ZEB1/E-cadherin, vimentin signaling in colon adenocarcinoma
Source: Cell Death Discov. 2021 Mar 26;7:61. doi: 10.1038/s41420-021-00427-x (PMC7998025; doi:10.1038/s41420-021-00427-x)

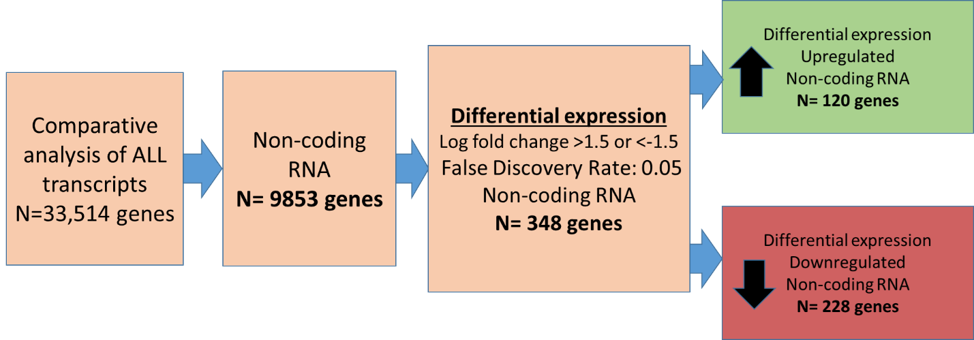

Supplement: Supplementary file 6 — Supplementary Figure 1 [file 41420_2021_427_MOESM6_ESM.tif]

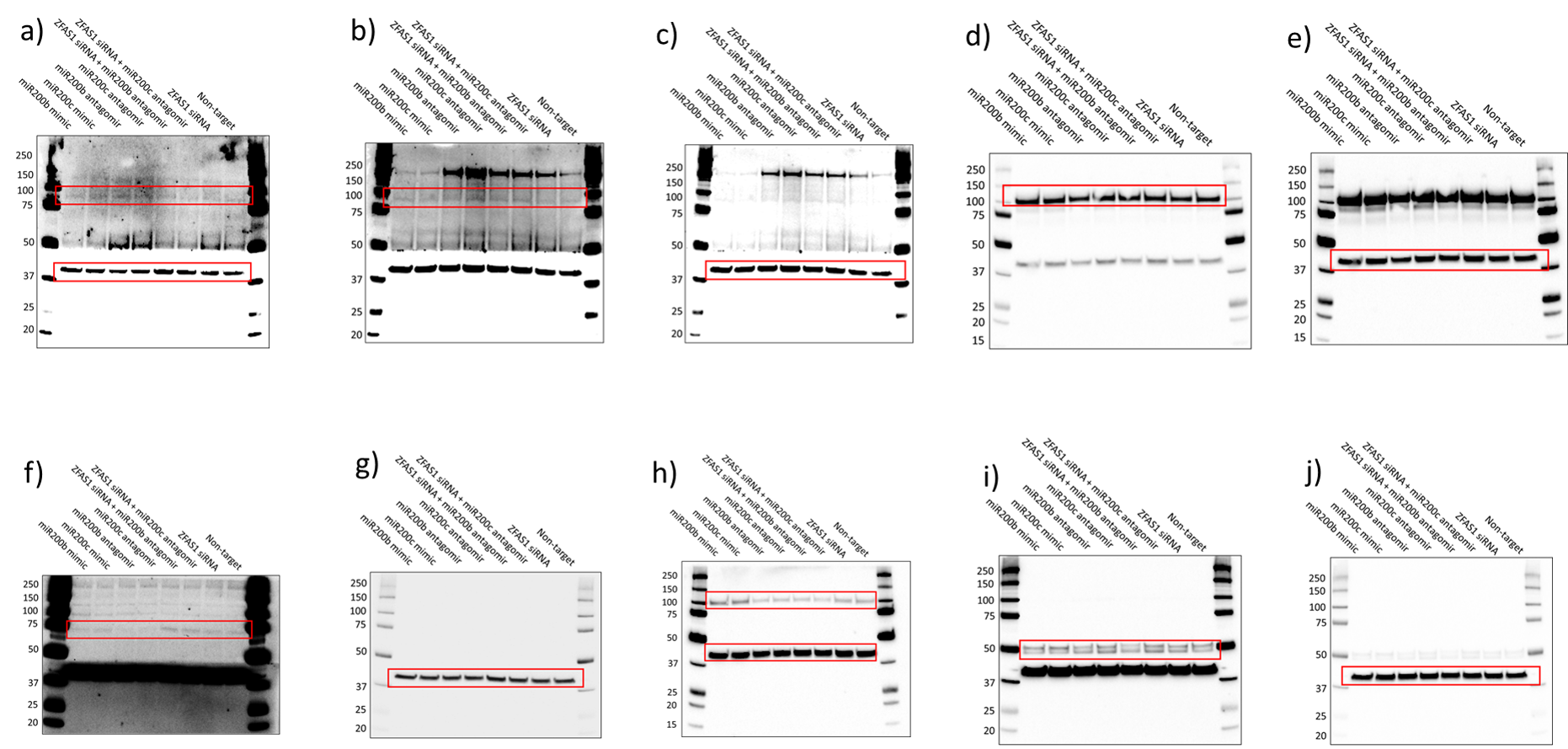

Supplement: Supplementary file 7 — Supplementary Figure 2 [file 41420_2021_427_MOESM7_ESM.tif]
