# Supplementary material for: Long non-coding RNA ZFAS1 is a major regulator of epithelial-mesenchymal transition through miR-200/ZEB1/E-cadherin, vimentin signaling in colon adenocarcinoma
Source: Cell Death Discov. 2021 Mar 26;7:61. doi: 10.1038/s41420-021-00427-x (PMC7998025; doi:10.1038/s41420-021-00427-x)
Supplement: Supplementary file 8 — Supplementary methods [file 41420_2021_427_MOESM8_ESM.docx]

**Methods:**

*Ethics statement*

This study was approved by the institutional review board of the University of Louisville. The in vivo experiments were approved by the Institutional Animal Care and Use Committee of the University of Louisville.

*The Cancer Genome Atlas and Gene Expression Omnibus (GEO)*

An application was made to the National Institute of Health Data Access Committee to obtain access to the raw sequencing data for the colon adenocarcinoma program. Patients with colon adenocarcinoma were identified from the Genomic Data Commons portal (https://portal.gdc.cancer.gov/). The raw sequencing data set was downloaded on February 21^st^ 2018 to the University of Louisville computer cluster, where it was encrypted until further use. The raw data was aligned to the most up-to-date human genome, using the ENSEMBL gene identifying codes (<https://useast.ensembl.org/index.html>). ). An R package “edgeR” was used to identify differentially expressed genes between a 40 patient subset that had both colon adenocarcinoma and normal colon epithelium RNA-seq data files available^37, 38^. A negative binomial generalized log-linear model was fitted to the read counts for each gene and likelihood ratio tests were conducted to identify differentially expressed genes.

The normalized gene expression and copy number of lncRNAs was downloaded using cBioPortal for Cancer (<https://www.cbioportal.org/>). The miRNA expression data of the colon adenocarcinoma patients was downloaded from FirebrowseR (Date: April 4^th^ 2018).

Three lncRNA expression data sets were downloaded from the GEO database. Two paired data sets (colon cancer tissue and normal colon mucosa from the same patient); GSE104836 (N=10) (Transcripts Per Kilobase Million (TPM) normalized) and GSE95132 (N=10) (Normalized RNA-seq expression) were downloaded, and one unpaired data set (colon cancer tissue and normal colon mucosa from different patients), GSE103512 (N=70) (Affymetrix microarray normalized expression) was downloaded. A sample size calculation was not performed for analysis using the COAD TCGA data set of the GEO data sets, but the data of all available patients was used for analysis.

*Bioinformatics analysis*

Each lncRNA was entered to the bioinformatics prediction program DIANA-lncBASE v2.0 (ref. 39). This bioinformatics tool allows for the identification of experimentally validated lncRNA-miRNA direct binding interactions. The ENSEMBL gene ID of each lncRNA was entered into the program and experimentally validated target miRNAs were generated for each lncRNA. lncRNA targets and signaling pathways were identified using Ingenuity Pathway Analysis (Qiagen, Hilden Germany.)

*Patient samples and Laser Capture Microdissection*

De-identified, fresh frozen samples of malignant colon adenocarcinoma and paired normal adjacent colon epithelium from 24 patients were obtained from the University of Louisville Surgical Biorepository. Informed consent was obtained from each patient for collection to the institutional biorepository. A specific sample size calculation was not used as this was an exploratory analysis in a limited number of patient samples. The normal tissue sample was taken 10cm from the tumor site. Tissue sections from these samples were cut at 7µm thickness and mounted on negatively charged slides from the Histogene™ LCM Frozen Section Staining Kit (Thermo Fisher Scientific, Waltham, MA). One slide per tissue sample was stained using hematoxylin and eosin for reference.

Tissue slides were individually stained using the Applied Biosystems Arcturus Staining Kit (Thermo Fisher Scientific, Bedford, MA). Immediately following slide staining, colon cancer or normal colon epithelium was identified by light microscopy at 4x and 10x magnification using the reference H&E slide. The ArcturusXT™ Laser Capture Microdissection System was used to isolate the tissue of interest onto CapSure® Macro LCM Caps (Thermo Fisher Scientific, Waltham, MA). RNA was extracted from the tissue on the caps using the PicoPure™ RNA Isolation Kit (Thermo Fisher Scientific, Waltham, MA), according to the manufacturers protocol.

*Cell lines*

The HT29 (ATCC® HTB-38™; Stage III), SW480 (ATCC® CCL-228™; Stage II), and Caco2 (ATCC® HTB-37™; Stage unknown) colon adenocarcinoma cell lines were purchased new from the American Type Culture Collection (Manassas, VA). The Cancer Cell Line Encyclopedia (https://portals.broadinstitute.org/ccle) is a Broad Institute (Boston, MA) database that contains RNA-seq expression data from common cancer cell lines. The encyclopedia was used to verify the expression of lncRNAs of interest in the selected cell lines. Gene mutations and the consensus molecular subtype of each cell line are previously described by Berg et al^12^. Cell lines were authenticated using Short Tandem Repeat profiling (ATCC Cell Line Authentication service, Manassas VA). This was performed every 6 months. Cell lines were tested for mycoplasma using the MycoFluor™ Mycoplasma Detection Kit (Life Technologies, Carlsbad, CA Reference: M7006).

*RNA interference*

Small interfering RNA (siRNA) were purchased from Dharmacon™ (Horizon Inspired Cell Solutions, Lafayette, CO). The Lincode™ siRNAs that were used are shown in **supplementary table 1**. Each siRNA was resuspended with a 1X siRNA buffer [Dharmacon™ (Horizon Inspired Cell Solutions, Lafayette, CO)]. Resuspended siRNAs were aliquoted in a working concentration and stored at -20°C. microRNA mimics (Dharmacon™ ,Horizon Inspired Cell Solutions, Lafayette, CO) and microRNA antagomirs (Life Technologies, Calrsbad, CA) were resuspended and stored at -20°C, per the manufacturers recommendations (**supplementary table 1**). All transfections were performed using Dharmafect 1 transfection reagent (Horizon Inspired Cell Solutions, Lafayette, CO).

*RNA extraction*

The Protein and RNA Isolation System*^TM^* (PARIS) kit (Life Technologies®, Carlsbad, CA) was used to separate and extract RNA from the nuclear and cytoplasmic compartments, per the manufacturers protocol. Total RNA extraction was performed using the Qiagen miRNeasy Mini kit (Qiagen, Hilden, Germany). RNA concentration and purity were assessed using Nanodrop® 2000 spectrophotometry (Thermo Fisher Scientific, Waltham, MA). For both RNA extraction protocols, RNA was considered pure and usable if the sample had a 260/280 ratio of 1.8-2.2. Samples were stored at -80°C until further use.

*Real time quantitative polymerase chain reaction (RT-qPCR)- mRNA and lncRNA quantification*

Complementary DNA (cDNA) was generated using the Superscript™ IV VILO™ Master Mix with ezDNase enzymes (Life Technologies, Carlsbad, CA). qPCR was performed using 1 µL of cDNA and TaqMan® mRNA assays (Life Technologies, Carlsbad, CA). Specific TaqMan® probes for RT-qPCR are shown in **Supplementary Table 2**. All reactions were completed in duplicate. Nucleic acid quantification was performed using a TaqMan Fast Advanced Master Mix (Life Technologies, Carlsbad, CA) on a Step-One Plus RT-qPCR system (Life Technologies, Carlsbad, CA). The expression levels of each of the genes were normalized to GAPDH using a cycle threshold (Ct) of 0.1 to calculate ΔCt values for analysis using the comparative ΔCt method^40^.

*miRNA quantification*

cDNA was generated from total RNA samples using the TaqMan® miRNA reverse transcription kit (Life Technologies, Carlsbad, CA). qPCR was performed using cDNA and TaqMan® microRNA assays (Life Technologies, Carlsbad, CA). Specific TaqMan® probes for RT-qPCR are shown in **Supplementary Table 2.** The expression levels of each miRNA were normalized to U6 using a cycle threshold (Ct) of 0.1 to calculate ΔCt values for analysis using the comparative ΔCt method^40^.

*Western blotting*

Following transfection, cells were lysed using radio immune-precipitation assay (RIPA) buffer and the protein concentration determined using the bicinchoninic acid (BCA) assay. The primary antibodies used are listed in **Supplementary Table 3**. To measure ZEB1 expression, 100 mcg of total protein from each cell lysate was loaded. For E-cadherin and Vimentin, 40 mcg of total protein was loaded. Each cell lysate was separated by NuPAGE^®^ MOPS SDS and transferred on to a nitrocellulose membrane. The membrane was incubated in equal parts of enhanced chemiluminescence (ECL) reagents A and B for 5 minutes in the dark to detect the target protein, and then developed and imaged using a ChemiDoc MP imager (BioRad, Hercules, CA).

*Cell proliferation and viability:*

HT29, SW480, and Caco2 cells were each plated at a concentration of 1x10^5^ cells/well in 2% Fetal Bovine Serum (FBS) supplemented media. The media was changed to 10% FBS supplemented media after 24 hours. Daily cell counts and percentage viable cells, using Trypan blue, were obtained at 24, 48, 72, 96 and 120 hours using a dual chamber cell slide (Bio-Rad Laboratories, Hercules, CA) and an automatic cell counter (TC20^TM^ Bio-Rad Laboratories, Hercules, CA).

*Scratch Assay*

HT29 and SW480 cells were plated at 1x10^6^ cells/well in a 12-well plate. Using a sterile 20μl pipette tip, a single vertical and horizontal scratch was made. A representative photo (Nikon Eclipse TS100 microscope at 4x magnification) was taken at the time of the initial scratch, as well as every 24 hours up to 120 hours or until complete scratch closure. The average distance between the scratch edges was calculated for each time point and the percentage closure for each time point was compared to 0-hours.

*Transwell Migration and Invasion*

Cells were re-suspended in serum-free media and pipetted into a transwell migration (COSTAR Transwell Permeable Supports, 8.0μm pore polycarbonate membrane), or invasion insert (COSTAR Transwell Permeable Supports, 8.0μm pore Corning BioCoat Matrigel Invasion Chambers). The matrigel invasion inserts were rehydrated for 2 hours with media prior to seeding cells. SW480 cells were seeded at 2 x 10^5^ cells/well, and HT29 cells were seeded at 5 x 10^5^ cells/well. Complete media (supplemented with 10% FBS) was used as a chemoattractant in each well. The plate was then incubated at 37°C for 24 hours. Non-migratory cells were removed with a cotton swab. Each insert was stained with a Modified Giemsa staining kit (Diff-Quik staining kit, Electron Microscopy Sciences, Hatfield, PA). Six pictures were taken of each insert at 10x magnification (Nikon Eclipse TS100,) and the migratory cells in each of these fields were manually counted.

*In vivo tumor growth*

Animal experiments were conducted in accordance with the ARRIVE guidelines. Six-week old male Nu/J mice were purchased from the Jackson Laboratory (Bar Harbor, Maine). There were no specific inclusion or exclusion criteria, or randomization techniques. SW480 cells were transfected with ZFAS1 siRNA or non-target siRNA. Four mice for each transfection condition were injected with cells. This sample size per group was determined from similar studies in the literature^39, 40^. A pair of mice for each condition were transfected at an individual time. Each mouse was injected with 5x10^6^ cells. Cells were resuspended in 200µL PBS at a final concentration of 2.5x10^7^. Each mouse was assessed every three days by two investigators (SOB, SAG). One investigator measuring the tumor volume was blinded to the transfection state (SAG). Tumor volume was calculated using the formula: Volume= 0.5 x longitude diameter x (latitudinal diameter)^2^. At 14 days post mass identification, the mice were sacrificed visa CO^2^ asphyixiation.

*Statistical analysis*

For each cell line and transfection condition, at least 5 replicates were performed, unless otherwise stated. All reactions were performed in duplicate, with the average being used for analysis. Data are presented as the mean ± standard deviation. The chi-squared test, or Fischer’s exact test, where appropriate, were used to compare categorical variables. The Mann-Whitney U test was used to compare continuous non-paired variables. The Wilcoxon signed-rank test was used to compare paired variables. The differences in overall survival were compared with Kaplan-Meier survival analysis and the Log Rank test. Statistical analysis was performed using SPSS v26.0 (IBM Corp, Armonk, NY). Graphs were created using GraphPad Prism v6.01 (Graphpad Software Inc. La Jolla, CA). Statistical significance was defined as p<0.05.

**References:**

39. Liang, W. C. et al. The LncRNA H19 promotes epithelial to mesenchymal transition by functioning as MiRNA sponges in colorectal cancer. Oncotarget 6, 22513–22525 (2015).

40. Jiang, H. et al. Long non-coding RNA SNHG15 interacts with and stabilizes transcription factor Slug and promotes colon cancer progression. Cancer Lett. 425, 78–87 (2018).
